# Peptide Nanofiber System for Sustained Delivery of Anti-VEGF Proteins to the Eye Vitreous

**DOI:** 10.3390/pharmaceutics15041264

**Published:** 2023-04-18

**Authors:** Seher Yaylaci, Erdem Dinç, Bahri Aydın, Ayse B. Tekinay, Mustafa O. Guler

**Affiliations:** 1 Faculty of Medicine, Lokman Hekim University, Ankara 06800, Turkey; 2 Department of Ophthalmology, Faculty of Medicine, Mersin University, Mersin 33000, Turkey; 3 Department of Ophthalmology, Faculty of Medicine, Gazi University, Ankara 06560, Turkey; 4 Requalite GmbH, Jahnplatz 4, 82166 Gräfelfing, Germany; 5 Pritzker School of Molecular Engineering, University of Chicago, 5640 S. Ellis Avenue, Chicago, IL 60637, USA

**Keywords:** ranibizumab, sustained drug release, hydrogels, peptide nanofiber, age-related macular degeneration

## Abstract

Ranibizumab is a recombinant VEGF-A antibody used to treat the wet form of age-related macular degeneration. It is intravitreally administered to ocular compartments, and the treatment requires frequent injections, which may cause complications and patient discomfort. To reduce the number of injections, alternative treatment strategies based on relatively non-invasive ranibizumab delivery are desired for more effective and sustained release in the eye vitreous than the current clinical practice. Here, we present self-assembled hydrogels composed of peptide amphiphile molecules for the sustained release of ranibizumab, enabling local high-dose treatment. Peptide amphiphile molecules self-assemble into biodegradable supramolecular filaments in the presence of electrolytes without the need for a curing agent and enable ease of use due to their injectable nature—a feature provided by shear thinning properties. In this study, the release profile of ranibizumab was evaluated by using different peptide-based hydrogels at varying concentrations for improved treatment of the wet form of age-related macular degeneration. We observed that the slow release of ranibizumab from the hydrogel system follows extended- and sustainable release patterns without any dose dumping. Moreover, the released drug was biologically functional and effective in blocking the angiogenesis of human endothelial cells in a dose-dependent manner. In addition, an in vivo study shows that the drug released from the hydrogel nanofiber system can stay in the rabbit eye’s posterior chamber for longer than a control group that received only a drug injection. The tunable physiochemical characteristics, injectable nature, and biodegradable and biocompatible features of the peptide-based hydrogel nanofiber show that this delivery system has promising potential for intravitreal anti-VEGF drug delivery in clinics to treat the wet form age-related macular degeneration.

## 1. Introduction

Age-related macular degeneration (AMD) is a common disease that may cause blindness in people over the age of 50 [1]. The incidence of this disease has been increasing due to the high percentage of middle-aged and older adults among the population, and it affects about 50 million people around the world [2]. The pathophysiology of AMD is not fully known. In the early stages of the disease, the accumulation of granules called drusen, which consist of a mixture of lipids, proteins, and minerals including cholesterol, phospholipids, triglycerides, complement factors, immunoglobulins, apolipoproteins, calcium, zinc, iron, glycosaminoglycans, extracellular matrix proteins, and inflammatory molecules, is observed in the Bruch’s membrane, which is considered residue from retinal pigment epithelium (RPE) cells. Since these accumulations rarely affect the photoreceptor layer of the retina, they usually do not cause any symptoms. However, the accumulation of drusen thickens the Bruch’s membrane, which causes ischemia in RPE cells. Ischemia instigates increased vascular endothelial growth factor (VEGF) secretion from the basal parts of RPE cells, and increased VEGF secretion triggers pathological angiogenesis in the choroid layer. Leakage from the new vessels in the choroid layer causes damage at the cellular level, and vision loss occurs [3,4]. Many studies have shown that choroid angiogenesis regresses with anti-VEGF agents [3]. Ranibizumab is the first anti-VEGF antibody approved for use in ophthalmology by the American Food and Drug Administration in 2006 [5]. Today, anti-VEGF antibodies are used in many eye diseases (diabetic retinopathy, premature retinopathy, etc.) in the pathophysiology of which VEGF plays a role [6,7]. Ranibizumab is a humanized monoclonal antibody fragment created using standard recombinant DNA technology and designed to fight against VEGF [5]. This fragment is one-third the size of the antibody and can easily pass through all layers of the retina when injected into the eye, and it binds to VEGF with high affinity [5]. Clinical studies have shown that the intravitreal use of ranibizumab is very effective and reliable in treating age-related macular degeneration [5,8]. The clinical protocol for using the drug is three doses of intravitreal injections that are one month apart [9,10]. The continuation of monthly injections after 3 months is decided according to the patient’s clinical, angiographic, and optical coherence tomography (OCT) findings. However, intravitreal drugs cannot stay in the vitreous for a long time due to the natural physiology of the eye, and they are eliminated quickly [6]. The available literature suggests that ranibizumab is eliminated from the body at a relatively slow rate, with a half-life ranging from 2.88 to 10.3 days, depending on the specific compartment measured. In addition, recurrent injections at short intervals may cause severe intraocular infections, especially endophthalmitis, and complications that can reduce the level of vision, such as retinal detachment, cataracts, and intraocular hemorrhage [3,4].

Currently, there are four different drug delivery techniques used for the posterior segment of the eye: topical, periocular, intraocular, and systemic [11]. In topical drug applications, sufficient drug concentrations could not be attained since the drug cannot freely pass into the intraocular tissues [12]. Similarly, in systemic applications, due to the existing blood–retinal barrier, there is not sufficient drug transition, and systemic side effects occur when the drug dose is increased. Intraocular (intravitreal) injection stands out among these drug administration techniques, since the drug is applied at the desired concentration, and treatment can be performed effectively; however, this method may cause various complications. Several studies have been carried out in recent years on the development of enhanced drug delivery systems using microspheres [13,14,15], polymeric materials [16,17,18], micelles [19,20,21,22], liposome systems [23,24], and hydrogels [24,25,26] in order to achieve the most effective concentration and the desired duration with the least invasive intervention. These systems aim to increase the half-life of ophthalmic drugs by preventing them from being easily removed. Delivery systems are able to maintain the drug concentration at a constant level and prevent the side effects that accompany the drug being administered with a high-dose single injection [27]. The drug’s molecular weight and lipophilic properties affect its half-life in the eye. Small-molecule drugs can be removed more easily from the vitreous part of the eye than larger ones. Therefore, when small molecular-weight anti-angiogenic drugs such as pegaptanib were coated with polyethylene glycol material, their intraocular half-life was prolonged [28]. Although such modifications increase the drug’s half-life, the length of the duration achieved through this method is insufficient considering the treatment duration of the disease. Therefore, intraocular transport and release systems that provide a longer drug half-life are needed. Non-degradable implants, which allow the drug to be released for an extended period, consist of a core section encapsulating the drug, a permeable membrane surrounding the drug (e.g., polyvinyl alcohol), and a semi-permeable membrane surrounding the whole system (e.g., silicone), and they are capable of constant drug release for at least one year [28]. Moreover, recently, an ocular implant (Susvimo) from Genentech acquired approval from the FDA, and it offers two treatments per year [29].

Biodegradable release systems can provide a more modular design with different shapes and sizes using different biocompatible materials that decompose into non-toxic substances when broken down by the body. In these systems, the drug release regime depends on the type of the polymer used, the morphological state in which it is injected, the amount of drug loaded, and the pore size of the system. Ozurdex^TM^ (formerly Posurdex^TM^; Allergan, Inc., Irvine, CA, USA), a carrier system used to treat retinal vascular blockages, has been approved by the American Food and Drug Administration and enables the long-term release of dexamethasone from polylactic glycolic acid [27]. Micro/nano-spheres for drug encapsulation have also been investigated as an alternative treatment method. In these systems, homogeneous distribution of the drug is achieved inside the microcapsules. Polylactic acid microspheres have been shown to enable a constant release for 1.5 months in a rabbit eye model [15]. Despite being injectable, these systems cause short-term temporary vision impairment following injection. Vision impairment passes when microspheres collapse into the lower part of the vitreous over time.

Although drug release systems ensure that the drug is protected from intraocular enzymes and released for an extended duration inside the eye, some transport systems have disadvantages such as loss of the structure and function of the drug when integrated with the carrier [30]. For antiangiogenic drugs that are made of proteins, integration into a carrier material is especially critical, since the protein structure is vital for its function and integration should not disrupt its structure.

Due to their low toxicity, excellent biocompatibility, and capacity to target small therapeutic drug molecules and proteins, supramolecular self-assembled peptide amphiphile nanostructures are a promising alternative to conventional delivery vehicles. Self-assembled peptide amphiphile molecules are a bottom-up way of creating scalable nanosized delivery systems. Molecular self-assembly is a viable method for spontaneously organizing molecules into ordered structures using free-energy processes such as van der Waals, electrostatic, hydrogen bonding, and π-π stacking interactions [31]. Peptide hydrogels also offer advantageous mechanical features, such as shear-thinning for easy distribution and extended-release patterns to maximize drug uptake while reducing overall drug dose. When employed to deliver therapeutics to the brain [32], eye [33,34], cardiovascular system [35], bone tissue [36], or cancer cells [37,38], self-assembled peptide nanostructures have exhibited promising results. Several studies have demonstrated the efficacy of PA-based delivery systems for anti-VEGF therapy in animal models of ocular diseases. For example, one study reported on the use of a PA-based hydrogel to deliver anti-VEGF proteins to the retina of rats with laser-induced choroidal neovascularization. The hydrogel was able to sustain the release of the anti-VEGF protein for up to 21 days and significantly reduced the size of the neovascular lesions compared to control groups. Another study reported the use of a PA-based nanofiber system to deliver anti-VEGF proteins to the vitreous of rabbits with experimental retinal neovascularization. The nanofiber system was able to sustain the release of the anti-VEGF protein for up to 28 days and significantly reduced the area of neovascularization compared to control groups [11].

In this present study, we utilized a biodegradable self-assembled peptide nanofiber hydrogel system for the sustained release of an anti-VEGF drug, ranibizumab. The negatively charged peptide amphiphile molecule (E-PA) spontaneously formed an injectable supramolecular hydrogel nanofiber with the ranibizumab drug solution. The resulting hydrogel nanofiber encapsulating ranibizumab provided extended-release with a single dose of the drug in vitro with no dose dumping or fluctuation over time. The drug level can be sustained and controlled within the specific dosage window over a significant period (between 10–150 h). Moreover, depending on the concentration of the peptide amphiphile molecules used in the formation of hydrogel nanofiber, the amount of encapsulated ranibizumab can be modulated easily. When a peptide solution with a higher molar concentration is used, the drug is entrapped in a tighter, more compact, and less porous hydrogel, thereby reducing the diffusion rate. On the other hand, when a peptide solution with a lower molar concentration is used, the drug diffuses from a highly porous hydrogel faster. In addition, drugs released from hydrogel nanofibers are fully functional as assessed by their effects on human umbilical vein endothelial cells (HUVECs) in vitro. In the in vivo study performed in the rabbit eye, the drug concentration remained higher for 7 days compared to the control group.

## 2. Materials and Methods

### 2.1. Materials

Wang resin, 9-Fluorenylmethoxycarbonyl (Fmoc) and tert-butoxycarbonyl (Boc)-protected amino acids, and 2-(1H-benzotriazol-1-yl)-1,1,3,3 tetramethyluronium hexaf luoro-phosphate (HBTU) for peptide amphiphile synthesis were purchased from NovaBiochem (Alexandria, NSW, Australia). Lauric acid and N,N- diisopropylethylamine (DIEA) were purchased from Merck (Rahway, NJ, USA). Other chemicals were purchased from Alfa Aesar (Haverhill, MA, USA) or Sigma-Aldrich (St. Louis, MI, USA) and used without any purification. We ensured that all chemicals were of analytical grade and were used without further purification.

Millipore Milli-Q deionized water were used during the experiments with a resistance of 18 MΩ·cm. Vascular endothelial growth factor (VEGF) was purchased from R&D Systems (293-VE-050/CF). The cell proliferation kit was purchased from Sigma (Cat. No. 11 465 007 001), and the goat-anti-human IgG/F (ab)2 antibody labeled with horseradish peroxidase was purchased from Thermo Fisher (Catalog #31122), Waltham, MA, USA.

All cell culture chemicals were purchased from Biological Industries (Kibbutz Beit Haemek, Israel), and cell culture consumables were purchased from Nest Scientific USA Inc. (Woodbridg, NJ, USA). Finally, the Lucentis used in our study was donated by Gazi University, Department of Ophthalmology.

### 2.2. Synthesis, Purification, and Characterization of Peptide Amphiphile Molecules

The peptide amphiphile molecule, E-PA, was synthesized using the Fmoc-protected solid-phase peptide synthesis method. E-PA was composed of an alkyl group, a β-sheet-forming peptide sequence, the VVAG, and a negatively charged amino acid, E. For synthesis, Rink amide resin was preloaded with 1.1 mmol/g Fmoc-Glu (OtBu)-OH, with a resultant concentration of 0.72 mmol/g. All amino acid couplings were performed with two equivalents of Fmoc-protected amino acid, 1.95 equivalents of hexafluorophosphate benzotriazole tetramethyl uronium (HBTU), and three equivalents of N,N-diisopropylethylamine (DIEA) in dimethylformamide (DMF) for 3 h. Fmoc deprotection was performed with 20% piperidine/DMF solution for 20 min. The cleavage of the peptides from the resin and deprotection of acid-labile protected amino acids was carried out through treatment with a mixture of trifluoroacetic acid (TFA):triisoproplysilane (TIS):water in the ratio of 95:2.5:2.5 for 2.5 h. Excess TFA was removed by rotary evaporation. The remaining residue was triturated with ice-cold diethyl ether, and the resulting white pellet was freeze-dried. The purity and molecular mass of the synthesized peptides were determined using the Agilent 6530–1200 QTOF LC-MS system with the addition of ESI-MS. Zorbax Extend-C18 2.1 × 50 mm columns were used for basic conditions. As a gradient, 0.1% TFA/water and 0.1% TFA/acetonitrile solutions were used. The purification was performed on a Zorbax Extend C18 prep-HT with a water/acetonitrile (0.1% NH_4_OH) gradient. Due to the acidic character of the E-PA, an Agilent 6530 accurate-Mass Q-TOF LC/MS was operated by eluting it from an Agilent Zorbax Extend-C18 (50 × 2.1 mm) column with a water/acetonitrile mixture (0.1% NH_4_OH) for the elucidation of the molecule. 

#### 2.2.1. Hydrogel Preparation

To obtain supramolecular hydrogels, E-PA dissolved in HEPES buffer (10 mM) was mixed with ranibizumab drug solution at the same volume at a specified ratio between 1:8 to 1:1. Since E-PA exhibited a negative charge at physiological pH, three-dimensional networks were formed when E-PA and the drug were mixed due to hydrogen bonding, van der Waals, and electrostatic forces. 

#### 2.2.2. Circular Dichroism (CD)

A Jasco J-815 CD spectrophotometer was used for CD analysis. Nanofiber formation was induced by mixing the drug and E-PA solutions, and the secondary structure of the mixture was spectrophotometrically monitored with a data range of 0.1 nm and a scan rate of 500 nm/min from 260 to 190 nm. The resulting raw data were converted to molar ellipticity. 

#### 2.2.3. Zeta Potential Analysis

Zeta potential measurements of E-PA, ranibizumab and hydrogel nanofibers were performed using a Malvern Zeta-ZS Zetasizer with 5 × 10^−5^ M at the indicated volume ratios.

#### 2.2.4. Rheology Measurements

The mechanical properties of the drug and E-PA mixture were examined by oscillatory rheology. Oscillatory rheology measurements were performed with an Anton Paar Physica MCR301. The viscoelastic behavior of the drug encapsulating hydrogel was determined by recording the storage (G′) and loss (G′′) moduli of the hydrogels for an hour at constant shear strain and angular frequency. A gap distance of 0.5 mm was used with an angular frequency of 10 rad/s and shear strain of 0.1%. All time-sweep experiments were carried out at room temperature. Measurements were performed with three replicates.

#### 2.2.5. Scanning Electron Microscopy

The morphological properties of nanofiber networks were examined through scanning electron microscopy with an FEI Quanta 200 FEG, using the GSED detector at ESEM mode with 3−10 keV beam energy. After 10 min of gelation on glass surfaces, the nanofibers were treated with gradually increasing ethanol concentrations and then dried using the Tousimis Autosamdri^®^-815b critical point dryer. The dried samples were coated with 3 nm Au/Pd and imaged with FEI Quanta 200 FEG SEM under a high vacuum.

### 2.3. Drug Encapsulation and Release Experiments

In the study, the preparation of hydrogels and drug-loaded hydrogels was carried out using serial dilutions of peptide solutions to obtain various hydrogel concentrations. The drug used in the experiments was ranibizumab, and its amount was kept constant at 10 mg/mL for all formulations. The 4% (*w*/*v*) E-PA solution was prepared in HEPES buffer at pH 7.4. To ensure homogeneity of the peptide solution, it was sonicated for 5 min. Then, serial dilutions of the stock peptide solution (4% (*w*/*v*) E-PA) were prepared to obtain different concentrations of 2%, 1%, 0.5%, 0.25%, 0.125%, and 0.0675% (*w*/*v*) peptide solutions.

To form the drug-loaded hydrogels, 10 µL of the E-PA solution, which was diluted from the stock solution (4% (*w*/*v*)), at different weight/volume ratios, was mixed with 10 µL of the ranibizumab drug solution. The resulting hydrogels were at weight ratios of 1:2, 1:1, 1:1/2, 1:1/4, and 1:1/8 of [Ranibizumab]: [PA]. After mixing, the hydrogels were incubated at 37 °C for 1 h to ensure hydrogel stability. Then, 50 µL of water was slowly added on top of each hydrogel, and the initial measurement was taken at t = 0.

In order to generate a standard curve, various ranibizumab concentrations were prepared, and the A280 protocol on the NanoDrop 2000c Spectrophotometer [39] was used to assess them (Figure S4). This approach is preferred, as it produces results in samples with a small volume (2 µL). Following the initial measurement at time zero, additional measurements were taken every hour for the first 10 h, and then measurements were performed every 8 h for three days. Three replicates of the measurements were made at room temperature.

Moreover, the release of ranibizumab from hydrogels was also achieved into the vitrein. Instead of water, 50 µL of vitrein was added onto the hydrogels containing the drug prepared in the ratios of 1:2, 1:1, and 1:1/2 as mentioned above, and samples were taken to measure the release of ranibizumab for 150 h in the same way. Measurements were performed as described in Section 2.7 using the ELISA protocol, unlike the other release study. Three replicates of the measurements were made at room temperature.

### 2.4. Enzymatic Degradation of Hydrogel Nanofiber

The enzymatic degradation of the peptide-based hydrogel nanofiber structure was also analyzed. E-PA was dissolved in 100 µL of double-distilled water to form a peptide solution at 10 µM concentration. To form nanofibers, nanofibers were formed by mixing drug solution (ranibizumab, 10 mg/mL) and E-PA (10 µM). A total of 750 µM rhodamine B (20 µL) was added to the E-PA solution to monitor the enzyme degradation while the PA molecules were mixed. After the nanofibers were prepared in quartz cuvettes, they were left to incubate for 3 h to equilibrate the formed nanofibers. Afterwards, 2.2 mL of proteinase K solution (1 mg/mL) or Tris buffer solution (10 mM) as a control was added onto the nanofibers (n = 3). The absorbances of the solutions were measured at room temperature between 400 and 600 nm with a Varian Cary 100 UV-Vis spectrophotometer at certain time intervals, including 0 h, 6 h, 12 h, 24 h, 48 h, 72 h, and 96 h.

### 2.5. Cellular Viability and Proliferation

The biocompatibility of the hydrogel nanofiber was monitored on two different cell lines: HUVECSs (ATCC: CRL-1730) and ARPE-19 (ATCC: CRL-2302™). The first set of experiments used ARPE-10 cells and aimed to understand the biocompatibility of hydrogel nanofiber. For the coating of culture plates, E-PA was dissolved in HEPES buffer (1 mM) and mixed with ranibizumab drug solution (10 mg/mL) at the same volume of E-PA to a total volume of 150 µL/cm^2^, and the gels were allowed to dry overnight under the hood. The coated cell culture plates were then sterilized by keeping them under UV for 30 min. For the ranibizumab group, 20 µL of ranibizumab (10 mg/mL) was added to growth medium of cells. At 24 h, 48 h, and 72 h after seeding, the cellular viability in all groups was determined according to the manufacturer’s protocol for the MTT (Sigma Aldrich, St. Louis, MI, USA) kit by normalizing spectrophotometric reads to ARPE-19 cultured on bare tissue culture plate (TCP) in 10% FBS. The cells used in the second series of cell culture studies were HUVECs. Prior to conducting an in vitro angiogenesis study using HUVECs, the purpose of this study was to assess the cell survival and proliferation variables, with the parameters of VEGF, FBS, and Matrigel. In this investigation, HUVECs cells were therefore cultivated in both Matrigel and non-Matrigel culture dishes. In order to understand the effect of VEGF on HUVECs’ cellular proliferation, cells were cultured with VEGF at varying concentrations. The fetal bovine serum (FBS) ratio was also changed so as to see its effect on HUVECs’ viability in other experiments. At 24 h after seeding, cellular proliferation in all groups was determined according to the manufacturer’s protocol for the MTT kit by normalizing spectrophotometric reads to HUVECs cultured on a bare tissue culture plate (TCP) in %15 FBS. Cellular viability was determined by staining HUVECs with Calcein AM (Invitrogen, Waltham, MA, USA). Prior to staining, 1250 cells/cm^2^ of HUVECs were seeded on Matrigel or TCP in similar mediums used for the proliferation experiment. At the 4th hour, the viable cells were imaged and counted using Image J software, and the results were normalized to HUVECs cultured on TCP in %15 FBS.

### 2.6. In Vitro Angiogenesis Model Analysis

HUVECs were cultured in standard cell culture conditions (5% CO_2_ and 37 °C) in DMEM containing 10% FBS and 1% penicillin–streptomycin. The vascular formation of HUVECs in the presence of VEGF was studied using the Matrigel system. 

First, the concentration-dependent effect of VEGF on in vitro angiogenesis model was studied. HUVECs seeded on Matrigel or TCP (negative control) were treated with VEGF at two different concentrations (10 ng/mL and 50 ng/mL). At 24 h after cell seeding, the number of sprouting formations was calculated from images taken from each well. In the second set of experiments, the drug functionality was studied, during which the drug was directly applied to HUVECs seeded on Matrigel or TCP at two different concentrations (1 ng/well and 10 ng/well) so as to observe the inhibition of the sprouting formation. The number of sproutings in each well was calculated as above. In the third set of experiments, the drug was encapsulated into the peptide amphiphile hydrogel system and applied to HUVECs cultured on Matrigel or TCP in order to observe the functionality of the released drug. 

As the last set of experiments for this section, the effect of the drug released from various hydrogel systems on HUVECs was investigated in an in vitro angiogenesis model. HUVECs were cultured in 24-well plates coated with Matrigel. In order to analyze the effect of released drug from peptide hydrogels, hydrogel was formed in an insert system so as to expose cells to drug-loaded hydrogels during culturing. Peptide solutions were prepared and sterilized under UV for 30 min. There were three experimental samples and four control samples. Experimental samples were the same except for their peptide concentrations in the gel in the insert system. E1: 10 µL drug + 10 µL peptide solution 1 (2%) (1:2); E2: 10 µL drug + 10 µL peptide solution 2 (1%) (1:1); E3: 10 µL drug + 10 µL peptide solution 3 (0.5%) (1:1/2). At the end of 24 h of culture, vascular tube formation was imaged under the microscope and quantified using Image J software on eight images taken from each culture plate well.

### 2.7. In Vivo Analysis

The Animal Ethics Committee of Gazi University approved all animal procedures. For in vivo tests, six white female/male New Zealand rabbits (mean weight, 2500 g; age, 12 weeks) were employed. In order to analyze the in vivo release kinetics of the delivery system, a nanofiber delivery system was applied to the vitreous chamber of New Zealand rabbits. Pupil dilation was achieved topically with one drop of phenylephrine (2.5%) and one drop of tropicamide (0.5%) just before the injection. General anesthesia was administered with ketamine hydrochloride (50 mg/kg) and xylazine hydrochloride (5 mg/kg), and topical anesthesia was administered with proparacaine (0.5%). After cleaning the eyelid and its surroundings with povidone–iodine, a sterile perforated cover was applied, and the lid was excluded with a sterile blepharostat immediately before Intravitreal injection. For 1 min, 10% povidine was placed on the cornea–conjunctiva surface, and povidine was cleaned off with 0.09% NaCl. All procedures were performed under sterile conditions. Then, the ranibizumab hydrogel system was applied intravitreally with a 30-gauge tip and insulin injector 3 mm behind the limbus in the upper temporal quadrant. Thirty microliters of physiological saline were mixed with 30 µL of ranibizumab (10 µg/µL) and injected into the right eyes of 6 rabbits; 30 µL of 4% (*w*/*v*) E-PA was mixed with 30 µL of ranibizumab (10 µg/µL) (Table 1) and injected into the left eyes of 6 rabbits. After this procedure, moxifloxacin (0.5%) was dripped 3 × 1 drops daily for one week, and the eyes of the animals were monitored daily in terms of inflammation and infection. The animals were sacrificed at the end of the experiment via intravenous injection of 100 mg/kg sodium pentobarbital. Both eyeballs were promptly enucleated and placed in a −80 °C freezer. On the day of the analysis, the vitreous was eviscerated from the eye (Figure S10). Before analysis, the frozen vitreous was weighed and then defrosted and solubilized in 1.0 mL of phosphate-buffered saline (PBS) on a rotator overnight at 4 °C in 1.0 mL of 1% bovine serum albumin in PBS. The samples were centrifuged at 2000 rpm for 10 min the next day. After centrifugation, the volume of the sample was measured. Before the immunoassay, all samples were diluted. The 165-amino acid variant of human recombinant VEGF (R&D Systems, 293-VE-010/CF) was immobilized on high-binding plates suitable for luminescent detection. The VEGF was put onto each plate as 100 µL/well for overnight incubation at 4 °C. Then, plates were washed three times with PBS and blocked for 4 h at 4 °C with 1% bovine serum albumin in PBS. A standard curve was generated using ranibizumab of known concentrations for each assay (Figure S9). A goat-anti-human IgG/F (ab)2 antibody (Thermo Fisher, Catalog #31122) conjugated with horseradish peroxidase was used to identify the bound ranibizumab. The tagged anti-humanized monoclonal IgG/F (ab)2 was diluted in the buffer at 1:20,000. The diluted secondary antibody was aliquoted at 100 L/well onto the VEGF plate and incubated for 45 min at room temperature with agitation. The plate was rinsed three times with 0.05% Tween 20/PBS after this incubation. According to the manufacturer’s instructions, the chemiluminescent signal was triggered using the luminol-based SuperSignal ELISA Pico Chemiluminescent Substrate (Pierce Biotechnology). The chemiluminescent signal was detected on a microplate reader (Molecular Devices, SpectraMax M Series) and acquired over a time frame of 0.1 s/well [39].

## 3. Results and Discussion

### 3.1. Peptide Nanofiber Hydrogel Formation and Characterization

**Figure 1:**
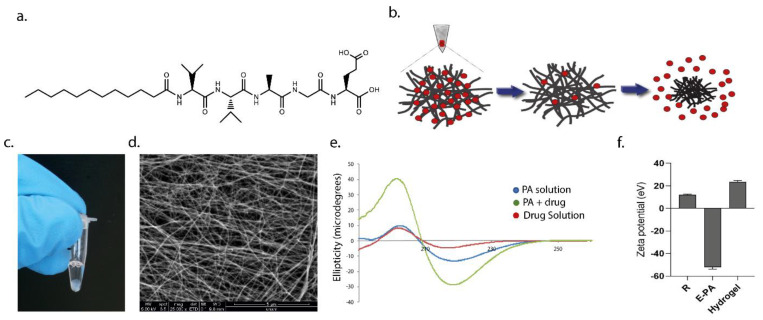
Peptide hydrogel nanofiber delivery system for the anti-VEGF drug. (**a**) Chemical structure of the glutamic acid-bearing peptide amphiphile (E-PA) molecule. (**b**) Schematic representation of anti-VEGF release strategy. (**c**) Self-supportive nanofiber delivery system formed in a tube. (**d**) SEM micrograph of the nanofiber network of E-PA/ranibizumab (1:1 [*w*/*v*]) (Scale bar = 5 µm). (**e**) Circular dichroism spectra of peptide amphiphile (E-PA) and its self-assembled nanofiber with ranibizumab (E-PA/ranibizumab). (**f**) Zeta potentials of ranibizumab, E-PA, and self-assembled nanofiber (E-PA/ranibizumab).

The peptide amphiphiles used in the study consist of alkyl chain, beta-sheet-forming sequence, intermediate amino acid, and bioactive sequence. Hydrophobic interactions play an important role in the formation of nanofibers up to micrometers in length by gathering PA molecules together. Hydrophobic interactions develop mostly between alkyl chains in nanofibers formed by PA molecules. Fatty acids with different carbon numbers (palmitic acid, oleic acid, lauric acid, caprylic acid, arachidonic acid, and linoleic acid) can be used as the alkyl chain. The carbon number of fatty acids affects the arrangement of peptide amphiphiles and, thus, their ability to form nanofibers and their flexibility to be rearranged. In this study, 12 carbon lauric acid was used to form the alkyl chain of the PA molecule. Hydrogen bonds are important when determining the tendency of PA molecules to form nanofibers or other structures. The amino acids in the beta-sheet-forming sequence are responsible for the establishment of hydrogen bonds between PA molecules. The intermediate amino acid and bioactive sequence vary according to the desired function to be imparted to the PA molecule [40]. The beta-sheet sequences of PA molecules to be used in the project were determined as double valine, alanine, and glycine, respectively. E-PA (Figure 1a) was synthesized by using the Fmoc solid-phase peptide synthesis method. The results of liquid chromatography (LC) and mass spectrometry (MS) on the synthesized peptides demonstrated that their molecular weights matched the theoretically calculated values, indicating that the peptides were synthesized successfully (Figure S1). The E-PA molecule bears a negative charge due to its glutamic acid residue, which acts as a charge neutralizer for self-assembly when mixed with Lucentis^®^ (ranibizumab). Since it does not carry any functional groups and is negatively charged, the E-PA molecule carrying only one glutamic acid was designed to form a hydrogel with ranibizumab. When E-PA was mixed with ranibizumab, the precipitation and gel formation started at weight concentrations as low as 1%. Non-covalent interactions such as hydrogen bonding, van der Waals, hydrophobic, and electrostatic interactions among the peptide amphiphile molecules and between the peptide amphiphile and drug molecules were the driving forces that promoted the self-assembly process and enabled the formation of the three-dimensional nanofiber networks [39,41,42] (Figure 1b). It is important to note that the buffer solution in which the drug is dissolved allows E-PA to self-assemble, because the drug is negatively charged in the first place. By neutralizing the charge of PA molecules with the charged groups in the buffer solutions, PA molecules change phase as has been extensively demonstrated in the literature [43].

The secondary structures of the self-assembled E-PA–ranibizumab nanofibers were studied by circular dichroism spectroscopy. As has been previously reported in the literature, nanofibers exhibit structural order during the self-assembly formation process [44]. PA molecules form fibers in the presence of opposite charges, and it is known that the dominant secondary structure in this fiber formation is the beta-sheet structure. Our aim with this study was to characterize the nanofiber formation of E-PA molecules mixed with drug solution. As is known in the literature, PA molecules can also gain fiber structure when mixed with buffer solutions. Therefore, the salts in the drug solution and the protein itself may be effective in nanofiber formation. The results revealed that these nanofibers were oriented in a beta-sheet conformation, displaying a negative minimum at 220 nm and positive ellipticity at 202 nm (Figure 1e). The received signal was minimal in the absence of drug solution or E-PA, but when they were combined, the signal was strong, suggesting the creation of nanofibers. During the formation of the nanofiber obtained in this result, whether the antibody proteins or the buffer solution is effective, it will be appropriate to conduct further analysis in a more detailed study. Moreover, zeta potential measurements further supported the establishment of the self-assembly process, because mixing E-PA molecules with opposing charges of drug solution reduced the stability of the individual solutions by 30 mV, confirming self-assembly aggregations at pH 7.4 (Figure 1f).

The E-PA–ranibizumab nanofibers were observed using scanning electron microscopy (SEM) and were observed to contain high aspect-ratio nanofibers with diameters to the order of 8–10 nm and lengths reaching several micrometers (Figure 1d). The morphology of the hydrogels formed by the E-PA–ranibizumab mixture exhibited a high-porosity nanofibrous network suitable for drug encapsulation and sustained release. 

The mechanical properties of the nanofibrous hydrogels (Figure S2) were examined by oscillatory rheology. A time sweep test was conducted by recording the storage (G′) and loss (G′′) moduli of the hydrogels for 1 h at constant shear strain and angular frequency in order to determine the viscoelastic behaviors of PA scaffolds. Oscillatory rheology measurements showed that the storage moduli were greater than the loss moduli for nanofiber hydrogels, suggesting that the nanofiber networks displayed solid elastic behavior (Figure S2). 

Furthermore, the biodegradability of hydrogel nanofibers was investigated. During gel production, the hydrogel nanofiber was infused with rhodamine B dye. After achieving gelation stability, the nanofibers were treated with proteinase K solution (1 mg/mL) or Tris buffer solution (10 mM) as a control. It was determined that the absorbance of rhodamine B increases with time (Figure S3). As evidenced by this increase in absorbance, this finding suggested that the presence of proteinase K degrades the nanofiber structures.

### 3.2. Peptide Nanofiber Hydrogel Formulations Enable Controlled Release

The drug encapsulation rates of hydrogel nanofibers were studied first. Nanofibers containing E-PA in different densities were used ([ranibizumab]:[PA], from 1:1/8 to 1:8) and examined the difference between them. Accordingly, nanofiber with the lowest E-PA ratio (1:1/8) gave the lowest encapsulation ratio (68.8 ± 0.4%, 68.8 µg [initial dose: 100 µg]) (Figure S5). As the ratio of E-PA in the nanofiber increased, the encapsulation ratio increased, reaching the highest point (97.6 ± 0.2%, 97.6 µg [initial dose: 100 µg]) for [ranibizumab]:[PA],1:2 nanofiber. If the [PA] ratio was increased further, the encapsulation efficiency decreased. At these rates, the hydrogel density probably decreased and the hydrogel stability deteriorated.

**Figure 2:**
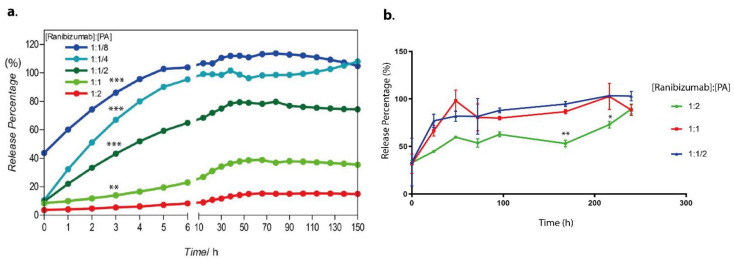
The cumulative release profile of the anti-VEGF ranibizumab from the peptide nanofiber gels with different [ranibizumab]:[PA] ratios (**a**) into water (from 1:1/8 to 1:8) and (**b**) into vitrein (from 1:1/2 to 1:2). Values represent mean ± SEM, n = 3 (*** *p* < 0.0001, ** *p* < 0.01, * *p* < 0.05).

Peptide–drug hydrogels were prepared at different concentrations in order to examine the release regimen of the drug from the hydrogel system and thereby determine the most appropriate gel formulation that maintains and releases the drug in a therapeutic range. Ranibizumab release from hydrogels at different peptide concentrations was monitored for 150 h. The concentration was measured spectrophotometrically at 280 nm (see Supporting Information). The release regimen was in biphasic mode for each hydrogel system. Depending on the concentration, 6–42% of the drug was released immediately after hydrogel formation. The rest was released more slowly with an extended-release over the following hours (Figure 2a). The extended-release mode showed that the hydrogels continued to release the drug at a constant rate. It is crucial to note that as the drug was encapsulated into denser PA hydrogels, its release was more sustained. The gel compositions of 1:1 and 2:1 ([ranibizumab]:[PA]) released drug over a more extended time frame as compared to gels having a lesser amount of PA (1:1/2–1:1/8). Specifically, the 1:1 gel released 28% of its drug load over the course of 150 h, and the 1:2 gel released 17% of its drug load over the course of 150 h. However, the 1:1/8 gel released 100% of its drug load in 3 h. This result clearly showed that the gel system was adjustable to modify the release regimen.

The amount of drug released could readily be adjusted by altering the amount of peptide amphiphile molecules utilized in the production of the hydrogel. The 1:2 [ranibizumab]:[PA] hydrogel was denser than the other hydrogel formulations, releasing almost 4.2% of the drug in a burst release and 18.8% of the drug over 150 h. The 1:1/8 [ranibizumab]:[PA] hydrogel, on the other hand, released roughly 43% of the drug in a burst release and released 100% of drug within 150 h. Low peptide content in the hydrogel system resulted in a reduced quantity of encapsulated drug molecules and a higher initial burst release; however, increasing the peptide concentration resulted in a higher-density network of nanofibers, which could block protein release and extend the total release time. This result demonstrated that peptide concentration adjustments allow for simple modulation of the amount of drug delivered over time (Figure 2a).

In addition to monitoring the amount of ranibizumab released into water, vitrein was also used in the release study. The release of ranibizumab from hydrogels placed in vitreous rather than water was monitored for 150 h by collecting samples at regular intervals. It was determined that, unlike in water, the hydrogel stability in vitreous degrades more rapidly, resulting in quicker drug release. Hydrogel nanofiber with a 1:2 formulation released 41.67% of the drug in the first 20 h and 98.7% by the end of 150 h. In less-dense hydrogels, this release is more rapid and reaches 100 percent more quickly. Hence, the hydrogel’s stability in vitreous must be optimized (Figure 2b).

### 3.3. In Vitro Analysis of the Functionality of the Drug Released from Peptide Nanofiber Hydrogels

**Figure 3:**
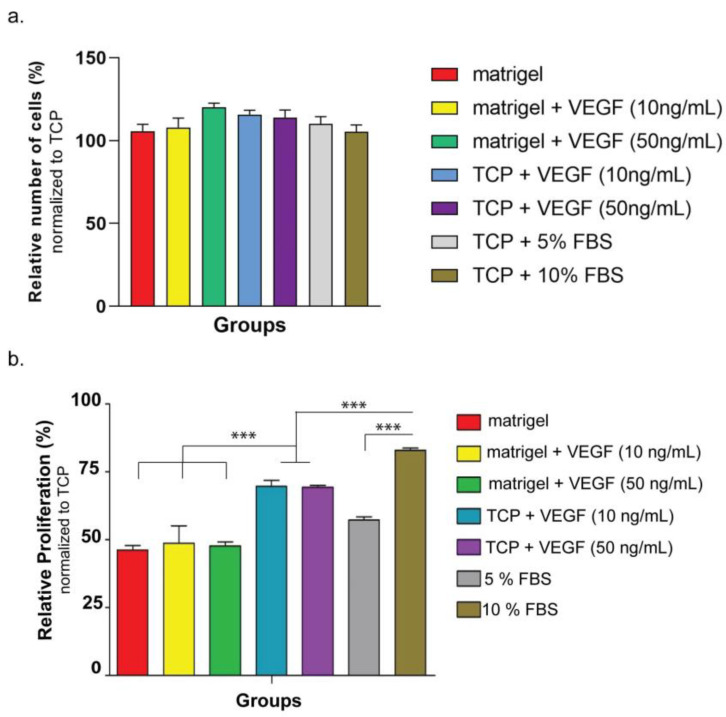
(**a**) Relative viability of HUVECs at 24 h cultured on Matrigel or bare tissue culture plate (TCP) with varying VEGF concentrations or FBS amount. The viability was normalized to HUVECs cultured on TCP with 15% FBS. (**b**) Relative number of HUVECs at 4 h cultured on Matrigel or TCP with varying VEGF concentrations or FBS amount. The average number of cells was normalized to HUVECs cultured on TCP with 15% FBS. Values represent mean ± SEM, n = 3 (*** *p* < 0.0001).

The functional activity of the released drug from the E-PA–ranibizumab hydrogel system was investigated by using HUVECs. Before this analysis, the biocompatibility of nanofiber hydrogel was investigated using ARPE-19 cells for three days. According to the results, on days 1, 2, and 3, the viability ratios of cells seeded on nanofiber coatings were comparable to the ratio of cells seeded on a bare tissue culture plate (TCP), indicating the biocompatibility of hydrogel nanofibers (Figure S6). Then, the cell viability and proliferation rate of HUVECs were examined when cultured on Matrigel or TCP with different concentrations of VEGF. After 4 h of culture, the cellular viability was observed to be similar for all groups when HUVECs were cultured on Matrigel compared to HUVECs cultured on TCP (Figure 3a and Figure S7). The cellular proliferation was evaluated after 24 h of culture, which revealed that the cells cultured on Matrigel divided more slowly than cells cultured on TCP, since HUVECs slowed proliferation when they were induced for angiogenesis on Matrigel (Figure 3b). Adding VEGF to the culture medium also induced HUVECs to go under vasculogenesis, and cells cultured in VEGF-containing medium on bare TCP had decreased proliferation rates compared to cells cultured without VEGF. Thus, both the addition of VEGF and culturing on Matrigel induced the angiogenesis mechanisms of HUVECs, and this induction resulted in a decrease of their proliferation rate.

Since ranibizumab is a protein drug, the integration of the drug into the delivery system is critical and should avoid damaging the structural conformity of the drug, which is directly related to its functionality. In order to analyze the functionality of the drugs released from nanofibers, an in vitro angiogenesis model was used. In this model, VEGF is supplemented to the culture medium of HUVECs seeded on Matrigel, which induces the HUVECs to form tubular structures. Matrigel is a mixture containing gelatin, laminin, and various proteins secreted by mouse tumor cells. Since HUVECs cultured in the presence of Matrigel form vascular structures within 24 h, this system is used as a model of angiogenesis in vitro. 

HUVECs were cultured on either Matrigel or TCP in the presence of two different VEGF concentrations or without VEGF. As shown in Figure S8, HUVECs formed tubular structures in the presence of VEGF compared to samples having no VEGF, where higher amounts of VEGF use resulted in a higher amount of sprouting and vice versa. As a result of these experiments, a 50 ng/mL VEGF concentration was chosen for the subsequent experiments as the optimal concentration for testing the effect of ranibizumab.

**Figure 4:**
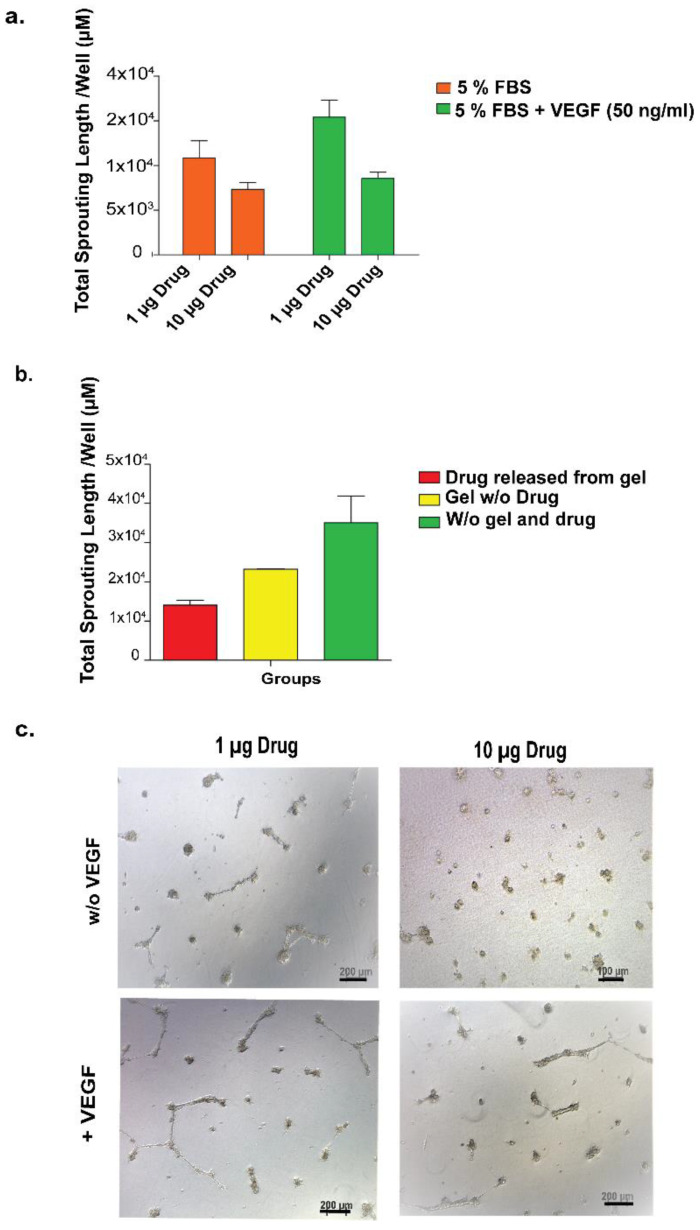
Anti-VEGF effect on HUVECs’ sprouting formation. (**a**) Tubular structure formation was inhibited in the presence of anti-VEGF, and this inhibition was in correlation with the drug concentration. (**b**) Light microscope images showing HUVECs on Matrigel with 1 µg or 10 µg anti-VEGF. (**c**) The number of total sprouting areas formed by HUVECs cultured on Matrigel at 48 h after they were seeded. HUVECs were exposed to drug released from the gel, gel without drug, or neither gel nor drug.

Next, we conducted an experiment to determine whether the inhibition effect of the drug correlates with its concentration. HUVECs were cultured on Matrigel in the presence or absence of VEGF (50 ng/mL) and were exposed to two different drug concentrations (1 ng/well and 10 ng/well), and the number of total sprouting formations was calculated. The results showed that HUVECs were responsive to the concentration change of the drug, and that at a higher concentration of drug, they demonstrated less sprouting both in the presence and the absence of VEGF (Figure 4a,b). It is also important to note that additional VEGF (50 ng/mL) had an additive effect on sprouting formation, as expected. 

Next, we tried to understand the functionality of the drug released from the gel system, and to do so, we conducted a release experiment analyzing the effect of the released drug on the formation of HUVECs’ sprouting when cultured on Matrigel. The results showed that the drug released from the hydrogel was able to inhibit growth factors and decrease sprouting formation when compared to no drug and no gel and drug control (Figure 4c).

**Figure 5:**
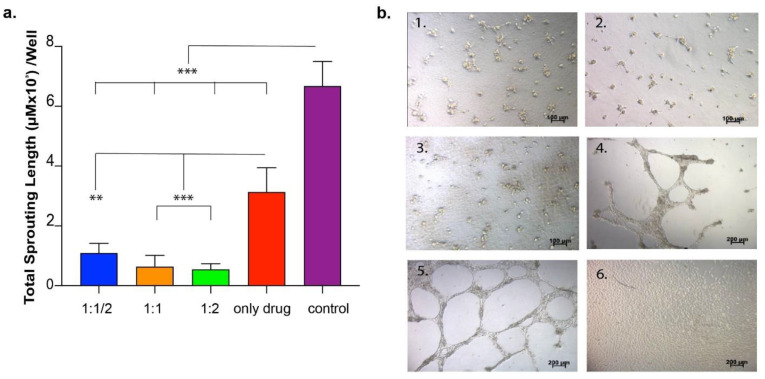
In vitro angiogenesis model used to evaluate released drug functionality. (**a**). The number of total sprouting areas formed by HUVECs cultured on Matrigel in the presence of VEGF. Cells cultured in culture media with anti-VEGF delivery systems ([ranibizumab]:[PA]; 1:1/2, 1:1, 1:2 experimental groups), drug only and on TCP (no drug, VEGF, peptide molecules and Matrigel); (**b**) Light microscope images of HUVECs cultured in (1) delivery system 1:1/2, (2) delivery system 1:1, (3) delivery system 1:2, (4) drug only, (5) w/o drug and delivery system, (6) TCP. Values represent mean ± SEM, n = 3 (*** *p* < 0.0001, ** *p* < 0.01).

Up to this point, it was shown that ranibizumab released from the hydrogel was able to inhibit the formation of the tubular structures and that this inhibition was correlated with the drug concentration. In order to obtain a modular drug-releasing system, different hydrogels prepared using different concentrations of E-PA were used and compared in terms of sprouting formation. For the experimental setup, nanofiber hydrogels were formed in an insert system in a cell culture plate-well in order to provide continuous drug release from nanofibers to the medium. At the end of 24 h, the total sprouting area was calculated for each well of the experimental and control groups. As shown in Figure 5, the drugs released from different nanofibers efficiently inhibited tube formation compared to non-drug controls. When different gels (1:1, 1:2, 1:1/2) were compared, the inhibition ratio correlated with the concentration of peptide amphiphile molecules in the nanofibers so that the more condensed nanofiber hydrogel induced less sprouting formation and vice versa (Figure 5). Moreover, hydrogels were more effective in inhibiting angiogenesis than the group where the drug was added in the absence of nanofibers. The overall result showed that the drug’s functionality was not reduced when encapsulated into the gel system. In addition, the gel system enhanced drug performance in terms of angiogenesis inhibition as compared to the drug-only groups.

### 3.4. In Vivo Analysis of Release Regimen of Peptide Nanofiber Hydrogel

**Figure 6:**
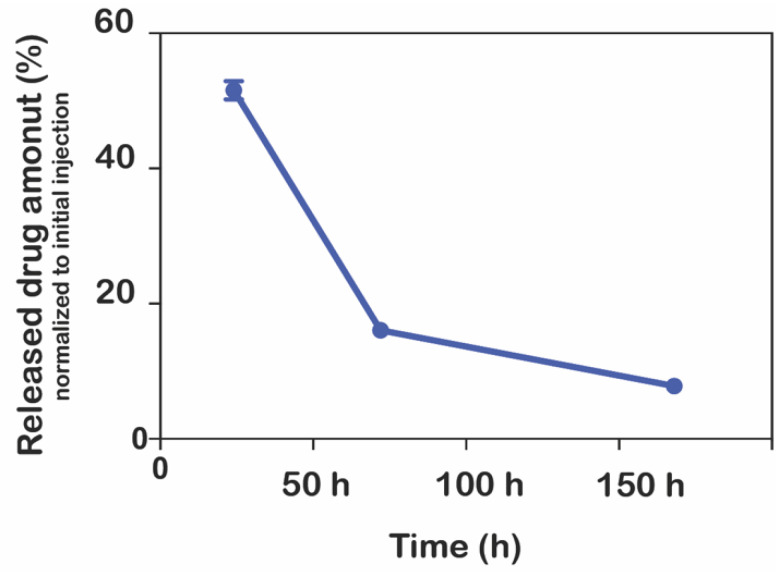
In vivo release profile of nanofiber gel [ranibizumab]:[PA]; 1:1. There was no detectable ranibizumab on day 7 in the control groups.

In order to observe the release kinetics of ranibizumab from peptide nanofibers in physiological conditions, nanofiber gels were applied to the vitreous chamber of the eyes of New Zealand rabbits and were monitored for seven days. According to release regimen in Figure 2, the gel at a 1:1 ratio was chosen to analyze release kinetics. No adverse effects, such as pain, irritation, eye redness, swelling, or vision guarding behavior, were observed in the animals. The vitreous was excised from the animals at the end of the experiments and analyzed in terms of ranibizumab concentration. The nanofiber system achieved a more sustained release of ranibizumab compared to control groups. The relative drug amount was found to be 15% normalized to the initial payload on day 7 (Figure 6). However, there was no detectable ranibizumab on day 7 in the control groups. This result indicated that there was a rapid initial release of the drug. This burst release was likely caused by drug molecules at or near the solvent–gel interface. The remaining drug would be released by bulk erosion of the gel system after an extended duration. Nevertheless, this study’s validity must be confirmed in an in vivo disease model that also addresses the released drug’s in vivo functionality.

## 4. Conclusions

In pathological conditions where the VEGF concentration rises in the vitreous chamber, the most commonly used treatment method is the injection of anti-VEGF antibodies into the eye’s posterior chamber. Although this treatment is effective and relieves the patient’s symptomatic complaints, the inability of the drug to remain at an effective dose until the disease regresses causes the patient to receive repetitive injections. Thus, there is a need for an extended-release system that does not affect drug function. In the current study, the anti-VEGF antibody, ranibizumab, was enclosed in an injectable peptide nanofiber hydrogel as a drug release platform. The fibrous character of peptide nanofiber hydrogels allows for a controlled drug release system which enables the drug concentration to stay within the therapeutic range. In addition, the hydrogel designed in this study allows for dose adjustment through a change in peptide concentration. In vitro cell culture studies revealed that the drug released from the system retains its biological function, suppressing the formation of vascular structures. Furthermore, the release kinetics of peptide nanofiber hydrogel were demonstrated in an in vivo model. Overall, we developed a biocompatible controlled anti-VEGF release system employing peptide amphiphile molecules, which will allow for patient-specific dose adjustments and a reduction in the patient’s required injection rate. The hydrogel structure will allow for a simple injection, will not affect the patient’s vision, and will biodegrade once the drug has been released.

## Figures and Tables

**Table 1:** Dosing regimen of rabbits to each eye for in vivo study.

	Ranibizumab	E-PA or Saline
Left Eyes	30 µL (10 µg/µL)	30 µL of 4% (*w*/*v*) E-PA
Right Eyes	30 µL (10 µg/µL)	30 µL of physiological saline

## Data Availability

The authors confirm that the data supporting the findings of this study are available within the article [and/or its Supplementary Materials].

## Supplementary Materials

The following supporting information can be downloaded at: https://www.mdpi.com/article/10.3390/pharmaceutics15041264/s1, Figure S1: (a) Reverse-HPLC chromatogram and (b) Liquid chromatography-mass spectrometry (LC-MS) analyses of E-PA; Figure S2: Rheology mesaurements showing (a) amplitude sweep and (b) frequency sweep of Ranibizumab:E-PA hydrogel; Figure S3: Enzymatic degradation of nanofiber; Figure S4: Standard curve of ranibizumab quantification through A280 protocol; Figure S5: Comparison of different [Ranibizumab]:[PA] groups in encapsulation efficiency; Figure S6: Viability analysis of ARPE-19 cells cultured on indicated platforms for day 1, 2 and 3. Results normalized to Day 1 TCP values; Figure S7: HUVECs cultured either on Matrigel or TCP with different VEGF concentrations stained with Calcein AM (Invitrogen) at 4 h (Scale bar; 50 nm); Figure S8: HUVECs cultured either on Matrigel or TCP with different VEGF concentrations; Figure S9: Standard curve of ranibizumab quantification through ELISA; Figure S10: Phases of in vivo experiment.

## References

[1] de Jong P.T.V.M. (2006). Age-related macular degeneration. N. Engl. J. Med..

[2] Saunier V., Bénédicte M.J.M., Delyfer M.-N., Cougnard-Grégoire A., Rougier M.-B., Amouyel P., Lambert J.-C., Dartigues J.-F., Korobelnik J.-F., Delcourt C. (2018). Incidence of and Risk Factors Associated With Age-Related Macular Degeneration: Four-Year Follow-up From the ALIENOR Study. JAMA Ophthalmol..

[3] Flores R., Carneiro Â., Vieira M., Tenreiro S., Seabra M.C. (2021). Age-Related Macular Degeneration: Pathophysiology, Management, and Future Perspectives. Ophthalmologica.

[4] Chen Y., Wiesmann C., Fuh G., Li B., Christinger H.W., McKay P., De Vos A.M., Lowman H.B. (1999). Selection and analysis of an optimized anti-VEGF antibody: Crystal structure of an affinity-matured fab in complex with antigen. J. Mol. Biol..

[5] Gaudreault J., Fei D., Rusit J., Suboc P., Shiu V. (2005). Preclinical Pharmacokinetics of Ranibizumab (rhuFabV2) after a Single Intravitreal Administration. Investig. Ophthalmol. Vis. Sci..

[6] Pożarowska D., Pożarowski P. (2016). The era of anti-vascular endothelial growth factor (VEGF) drugs in ophthalmology, VEGF and anti-VEGF therapy. Cent. Eur. J. Immunol..

[7] van der Giet M., Cornelia H., Mirjam S., Markus T. (2015). Anti-VEGF Drugs in Eye Diseases: Local Therapy with Potential Systemic Effects. Ingenta Connect..

[8] Steinbrook R. (2006). The Price of Sight—Ranibizumab, Bevacizumab, and the Treatment of Macular Degeneration. N. Engl. J. Med..

[9] Li E., Donati S., Lindsley K.B., Krzystolik M.G., Virgili G. (2020). Treatment regimens for administration of anti-vascular endothelial growth factor agents for neovascular age-related macular degeneration. Cochrane Database Syst. Rev..

[10] Horner F., Lip P.L., Mohammed B.R., Fusi-Rubiano W., Gokhale E., Mushtaq B., Chavan R. (2021). Comparing Effectiveness of Three Different Anti-VEGF Treatment Regimens for Neovascular Age-Related Macular Degeneration: Two Years&rsquo; Real-World Clinical Outcomes. Clin. Ophthalmol..

[11] Seah I., Zhao X., Lin Q., Liu Z., Su S.Z.Z., Yuen Y.S., Hunziker W., Lingam G., Loh X.J., Su X. (2022). Use of biomaterials for sustained delivery of anti-VEGF to treat retinal diseases. Eye.

[12] Ahmed I., Patton T.F. (1985). Importance of the noncorneal absorption route in topical ophthalmic drug delivery. Investig. Ophthalmol. Vis. Sci..

[13] Liu W., Borrell M.A., Venerus D.C., Mieler W.F., Kang-Mieler J.J. (2019). Characterization of Biodegradable Microsphere-Hydrogel Ocular Drug Delivery System for Controlled and Extended Release of Ranibizumab. Transl. Vis. Sci. Technol..

[14] Osswald C.R., Kang-Mieler J.J. (2016). Controlled and Extended In Vitro Release of Bioactive Anti-Vascular Endothelial Growth Factors from a Microsphere-Hydrogel Drug Delivery System. Curr. Eye Res..

[15] Moritera T., Ogura Y., Yoshimura N., Honda Y., Wada R., Hyon S.H., Ikada Y. (1992). Biodegradable microspheres containing adriamycin in the treatment of proliferative vitreoretinopathy. Investig. Ophthalmol. Vis. Sci..

[16] Park D., Shah V., Rauck B.M., Friberg T.R., Wang Y. (2013). An Anti-angiogenic Reverse Thermal Gel as a Drug-Delivery System for Age-Related Wet Macular Degeneration. Macromol. Biosci..

[17] Liu J., Li S., Li G., Li X., Yu C., Fu Z., Li X., Teng L., Li Y., Sun F. (2019). Highly bioactive, bevacizumab-loaded, sustained-release PLGA/PCADK microspheres for intravitreal therapy in ocular diseases. Int. J. Pharm..

[18] Lee S.S., Hughes P., Ross A.D., Robinson M.R. (2010). Biodegradable Implants for Sustained Drug Release in the Eye. Pharm. Res..

[19] Prima G.D., Saladino S., Bongiovì F., Adamo G., Ghersi G., Pitarresi G., Giammona G. (2017). Novel inulin-based mucoadhesive micelles loaded with corticosteroids as potential transcorneal permeation enhancers. Eur. J. Pharm. Biopharm..

[20] Li C., Chen R., Xu M., Qiao J., Yan L., Guo X.D. (2018). Hyaluronic acid modified MPEG-*b*-PAE block copolymer aqueous micelles for efficient ophthalmic drug delivery of hydrophobic genistein. Drug Deliv..

[21] Zhao X., Seah I., Xue K., Wong W., Tan Q.S.W., Ma X., Lin Q., Lim J.Y.C., Liu Z., Parikh B.H. (2021). Antiangiogenic Nanomicelles for the Topical Delivery of Aflibercept to Treat Retinal Neovascular Disease. Adv. Mater..

[22] Ideta R., Tasaka F., Jang W.-D., Nishiyama N., Zhang G.-D., Harada A., Yanagi Y., Tamaki Y., Aida T., Kataoka K. (2005). Nanotechnology-Based Photodynamic Therapy for Neovascular Disease Using a Supramolecular Nanocarrier Loaded with a Dendritic Photosensitizer. Nano Lett..

[23] Mu H., Wang Y., Chu Y., Jiang Y., Hua H., Chu L., Wang K., Wang A., Liu W., Li Y. (2018). Multivesicular liposomes for sustained release of bevacizumab in treating laser-induced choroidal neovascularization. Drug Deliv..

[24] Abrishami M., Ganavati S.Z., Soroush D., Rouhbakhsh M., Jaafari M.R., Malaekeh-Nikouei B. (2009). Preparation, characterization, and in vivo evaluation of nanoliposomes-encapsulated bevacizumab (avastin) for intravitreal administration. Retina.

[25] Bisht R., Jaiswal J.K., Chen Y.-S., Jin J., Rupenthal I.D. (2016). Light-responsive *in situ* forming injectable implants for effective drug delivery to the posterior segment of the eye. Expert Opin. Drug Deliv..

[26] Gaudana R., Jwala J., Boddu S.H.S., Mitra A.K. (2008). Recent Perspectives in Ocular Drug Delivery. Pharm. Res..

[27] Kuno N., Fujii S. (2011). Recent Advances in Ocular Drug Delivery Systems. Polymers.

[28] Bourges J., Bloquel C., Thomas A., Froussart F., Bochot A., Azan F., Gurny R., BenEzra D., Behar-Cohen F. (2006). Intraocular implants for extended drug delivery: Therapeutic applications. Adv. Drug Deliv. Rev..

[29] Protected Health Information (2021). WARNING: ENDOPHTHALMITIS The SUSVIMO implant has been associated with a 3-fold higher rate of endophthalmitis than monthly intravitreal injections of ranibizumab. Many of these events were associated with conjunctival retractions or erosions. Appropria.

[30] Ilochonwu B.C., Urtti A., Hennink W.E., Vermonden T. (2020). Intravitreal hydrogels for sustained release of therapeutic proteins. J. Control. Release.

[31] Lim J., Lin Q., Xue K., Loh X. (2019). Recent advances in supramolecular hydrogels for biomedical applications. Mater. Today Adv..

[32] Mazza M., Notman R., Anwar J., Rodger A., Hicks M., Parkinson G., McCarthy D., Daviter T., Moger J., Garrett N. (2013). Nanofiber-Based Delivery of Therapeutic Peptides to the Brain. ACS Nano.

[33] Lin Q., Lim J.Y., Xue K., Su X., Loh X.J. (2021). Polymeric hydrogels as a vitreous replacement strategy in the eye. Biomaterials.

[34] Xu X.-D., Liang L., Chen C.-S., Lu B., Wang N.-L., Jiang F.-G., Zhang X.-Z., Zhuo R.-X. (2010). Peptide Hydrogel as an Intraocular Drug Delivery System for Inhibition of Postoperative Scarring Formation. ACS Appl. Mater. Interfaces.

[35] Guo H.-D., Cui G.-H., Yang J.-J., Wang C., Zhu J., Zhang L.-S., Jiang J., Shao S.-J. (2012). Sustained delivery of VEGF from designer self-assembling peptides improves cardiac function after myocardial infarction. Biochem. Biophys. Res. Commun..

[36] Sutton S., Campbell N.L., Cooper A.I., Kirkland M., Frith W., Adams D.J. (2009). Controlled Release from Modified Amino Acid Hydrogels Governed by Molecular Size or Network Dynamics. Langmuir.

[37] Liu J., Xu H., Yang C., Zhang Y., Chu L., Liu J., Liu Q., Song N. (2013). Novel tumor-targeting, self-assembling peptide nanofiber as a carrier for effective curcumin delivery. Int. J. Nanomed..

[38] Fung S.Y., Yang H., Chen P. (2008). Sequence Effect of Self-Assembling Peptides on the Complexation and In Vitro Delivery of the Hydrophobic Anticancer Drug Ellipticine. PLoS ONE.

[39] Bakri S.J., Snyder M.R., Reid J.M., Pulido J.S., Ezzat M.K., Singh R.J. (2007). Pharmacokinetics of intravitreal ranibizumab (Lucentis). Ophthalmology.

[40] Niece K.L., Hartgerink J.D., Donners J.J.J.M., Stupp S.I. (2003). Self-Assembly Combining Two Bioactive Peptide-Amphiphile Molecules into Nanofibers by Electrostatic Attraction. J. Am. Chem. Soc..

[41] Hartgerink J.D., Beniash E., Stupp S.I. (2001). Self-assembly and mineralization of peptide-amphiphile nanofibers. Science.

[42] Diaferia C., Rosa E., Balasco N., Sibillano T., Morelli G., Giannini C., Vitagliano L., Accardo A. (2021). The Introduction of a Cysteine Residue Modulates The Mechanical Properties of Aromatic-Based Solid Aggregates and Self-Supporting Hydrogels. Chem.-A Eur. J..

[43] Paramonov S.E., Jun H.-W., Hartgerink J.D. (2006). Self-Assembly of Peptide-Amphiphile Nanofibers: The Roles of Hydrogen Bonding and Amphiphilic Packing. J. Am. Chem. Soc..

[44] Zhang S., Holmes T., Lockshin C., Rich A. (1993). Spontaneous assembly of a self-complementary oligopeptide to form a stable macroscopic membrane. Proc. Natl. Acad. Sci. USA.

