# Supplementary material for: Peptide Nanofiber System for Sustained Delivery of Anti-VEGF Proteins to the Eye Vitreous
Source: Pharmaceutics. 2023 Apr 18;15(4):1264. doi: 10.3390/pharmaceutics15041264 (PMC10141348; doi:10.3390/pharmaceutics15041264)
Supplement: Supplementary file 1 [file pharmaceutics-15-01264-s001.zip › pharmaceutics-2279307-supplementary.pdf]

Supporting Information for

Peptide Nanofiber System for Sustained Delivery of Anti-VEGF Proteins to the Eye Vitreous

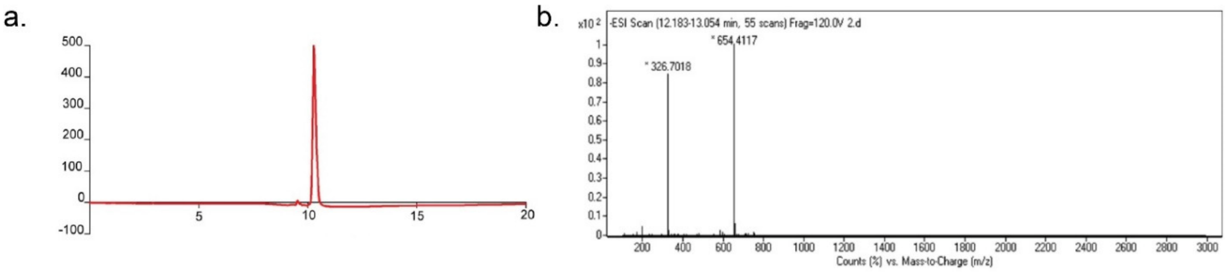

Figure S1 (a) Reverse-HPLC chromatogram and **(b)** Liquid chromatography-mass spectrometry (LC-MS) analyses of E-PA

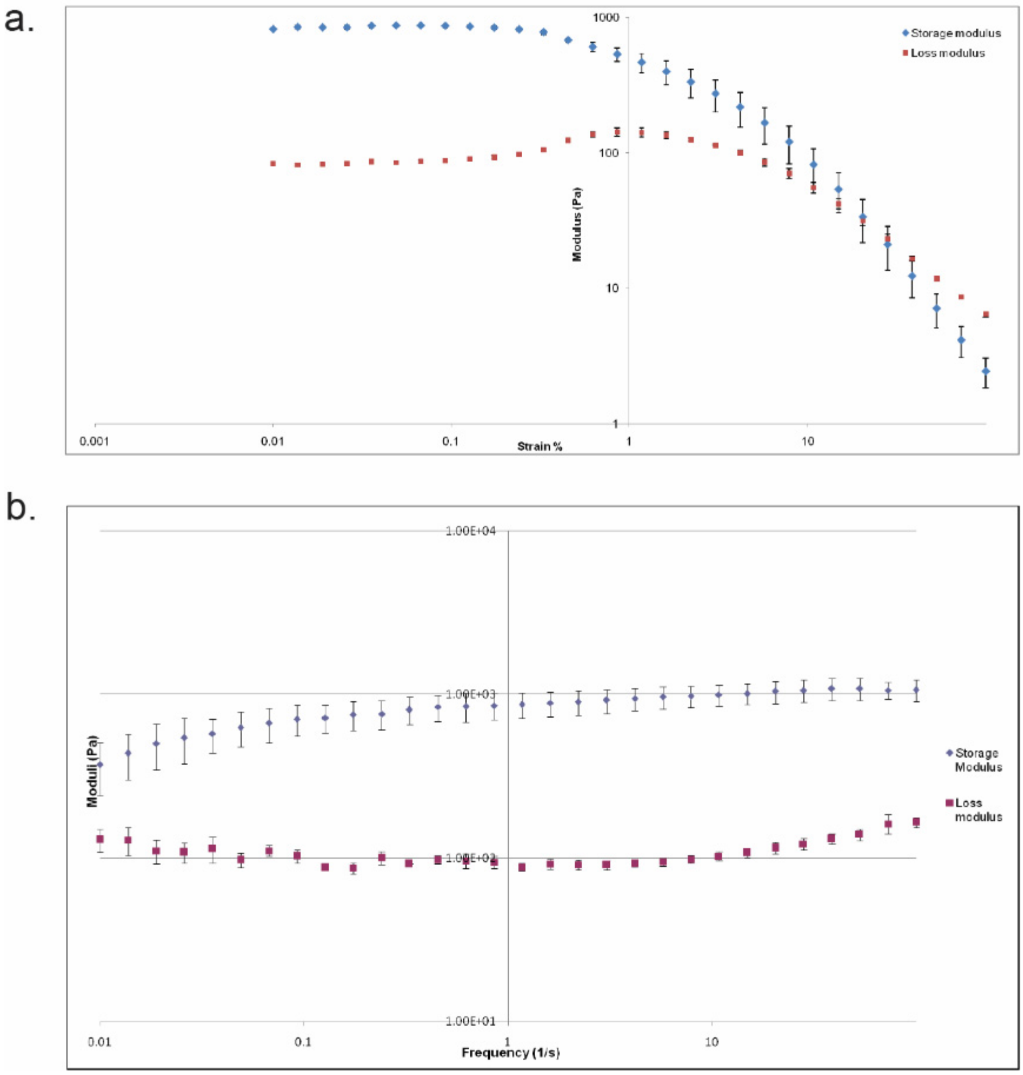

Figure S2 Rheology mesaurements showing **(a)** amplitude sweep and **(b)** frequency sweep of Ranibizumab:E-PA hydrogel

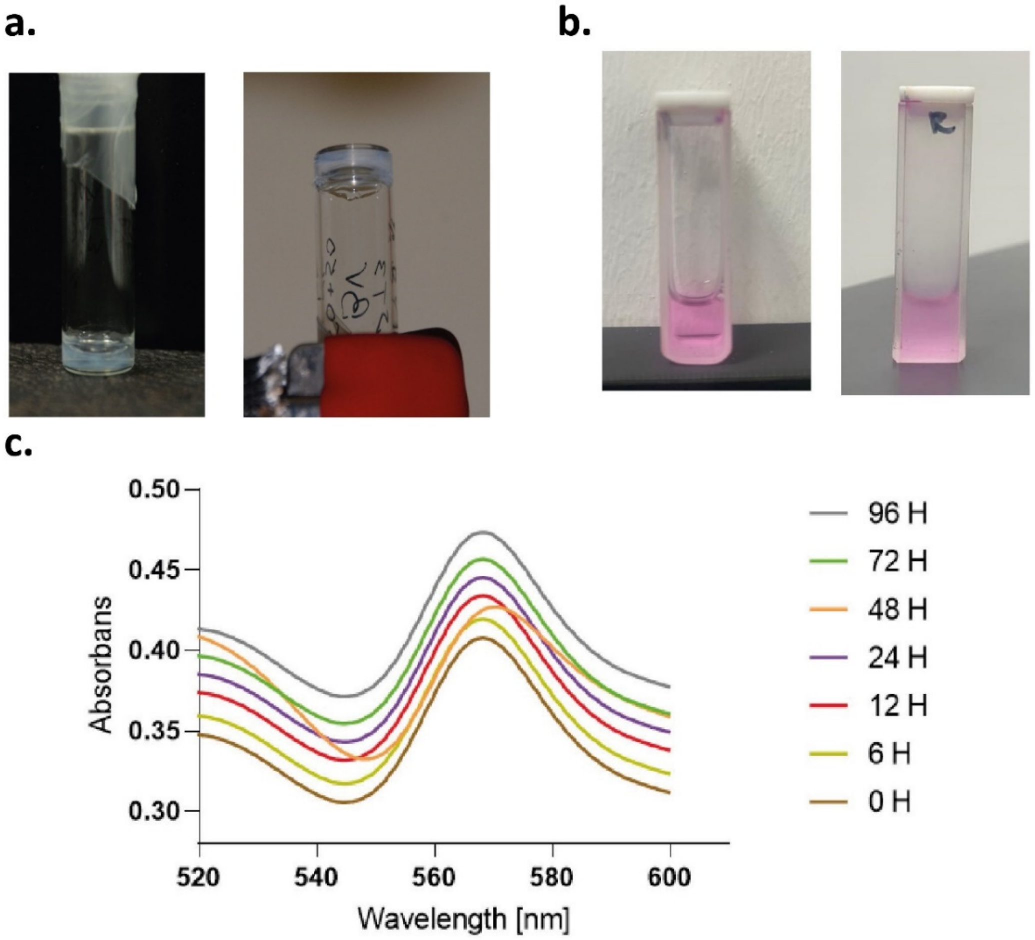

Figure S3 Enzymatic degradation of nanofiber. **(a)** Image of the formed nanofiber. **(b)** The image of the nanofiber mixed with the marker molecule Rhodamin B at t=0h (first image) and at t=96 h(second image) **(c)** The absorbance of the nanofiber containing Rhodamin B in the range of 0-96 hours following incubation with Proteinase K enzyme.

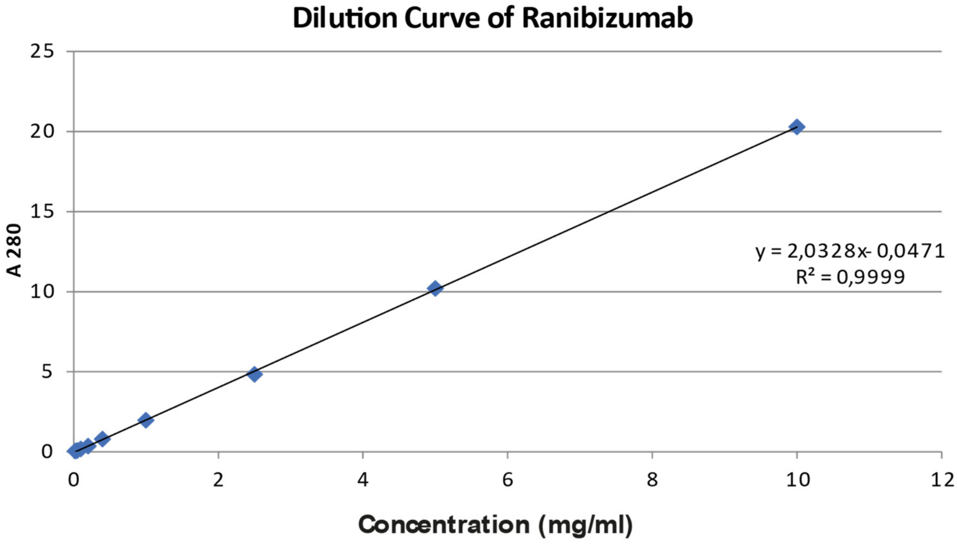

Figure S4. Standard curve of ranibizumab quantification through A280 protocol

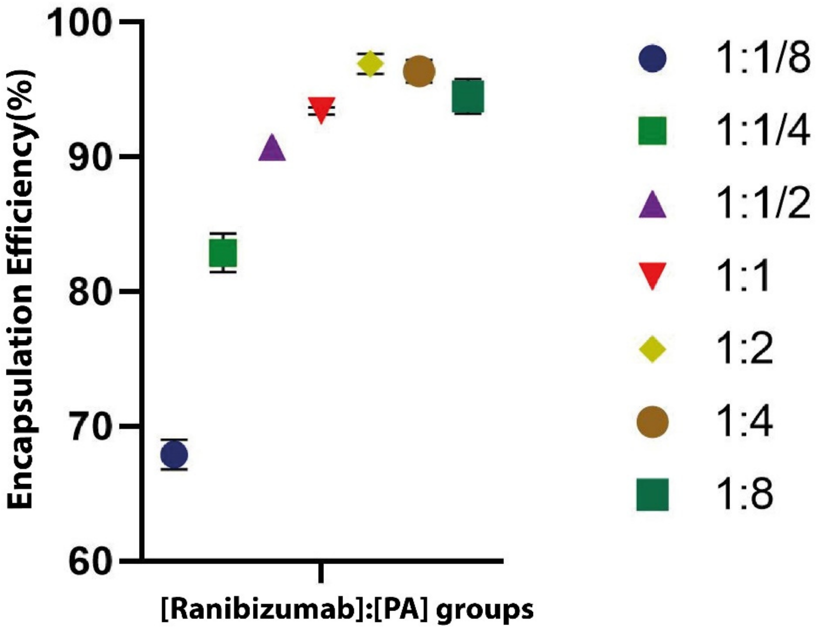

Figure S5. Comparison of different [Ranibizumab]:[PA] groups in encapsulation efficiency

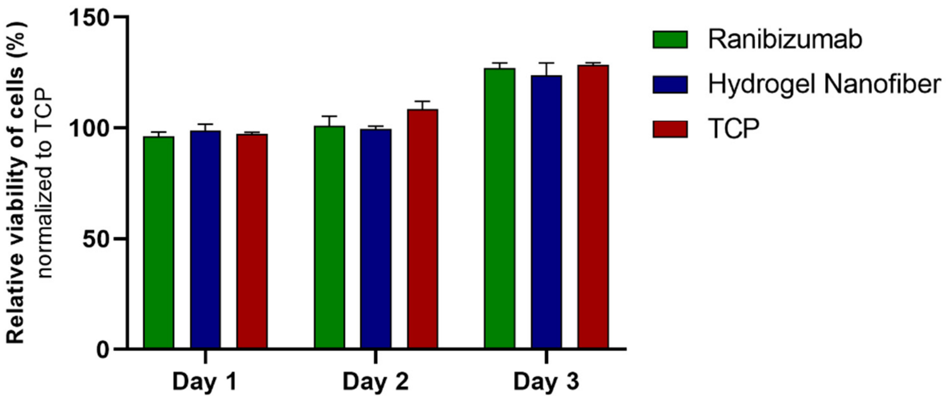

Figure S6. Viability analysis of ARPE-19 cells cultured on indicated platforms for day 1, 2 and 3. Results normalized to Day 1 TCP values.

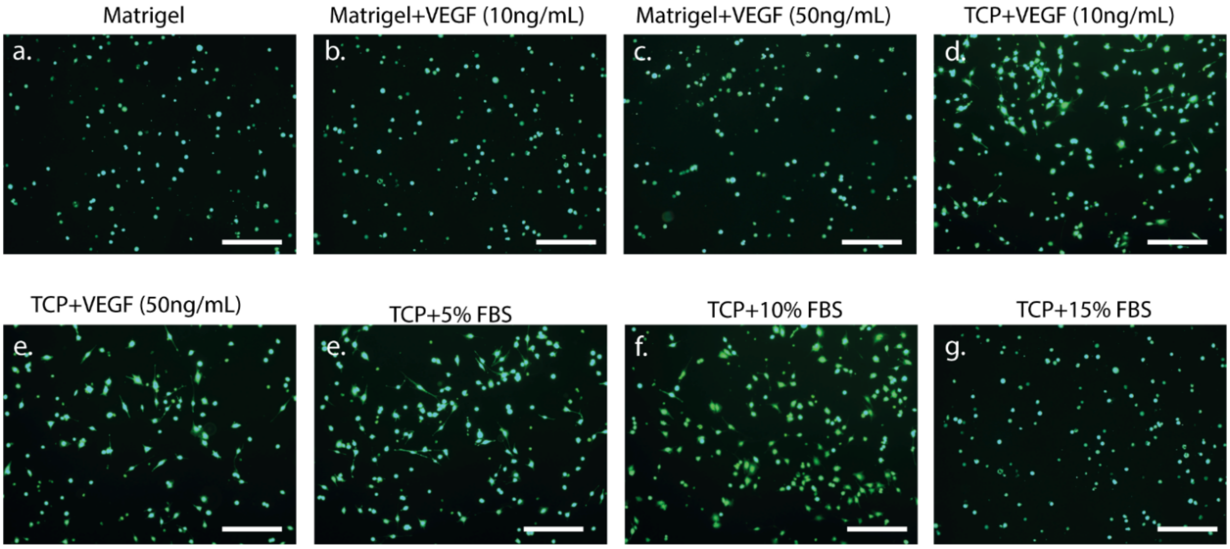

Figure S7 HUVECs cultured either on Matrigel or TCP with different VEGF concentrations stained with Calcein AM (Invitrogen) at 4 h (Scale bar; 50 nm)

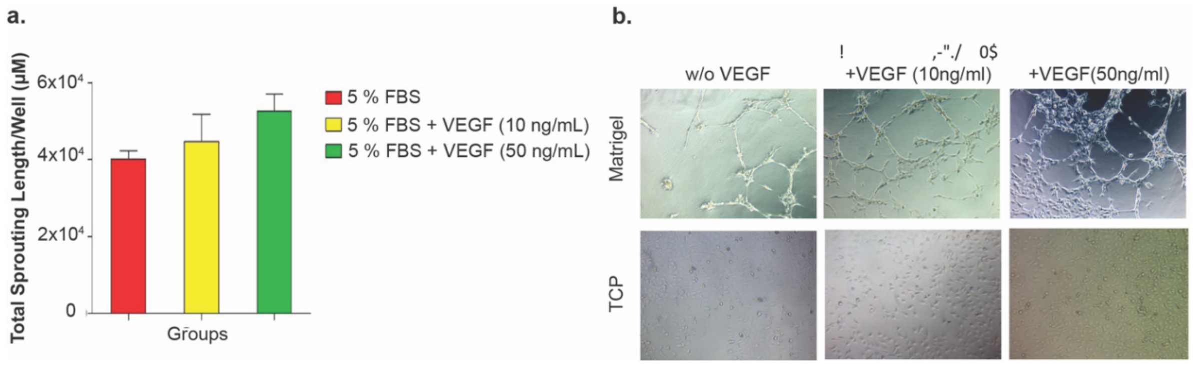

Figure S8 HUVECs cultured either on Matrigel or TCP with different VEGF concentrations. (a) The number of total sprouting area formed by HUVECs cultured on Matrigel in the absence of VEGF and in the presence of VEGF (10 ng/mL and 50 ng/mL). (b) Light microscope images of HUVECs cultured on Matrigel or TCP.

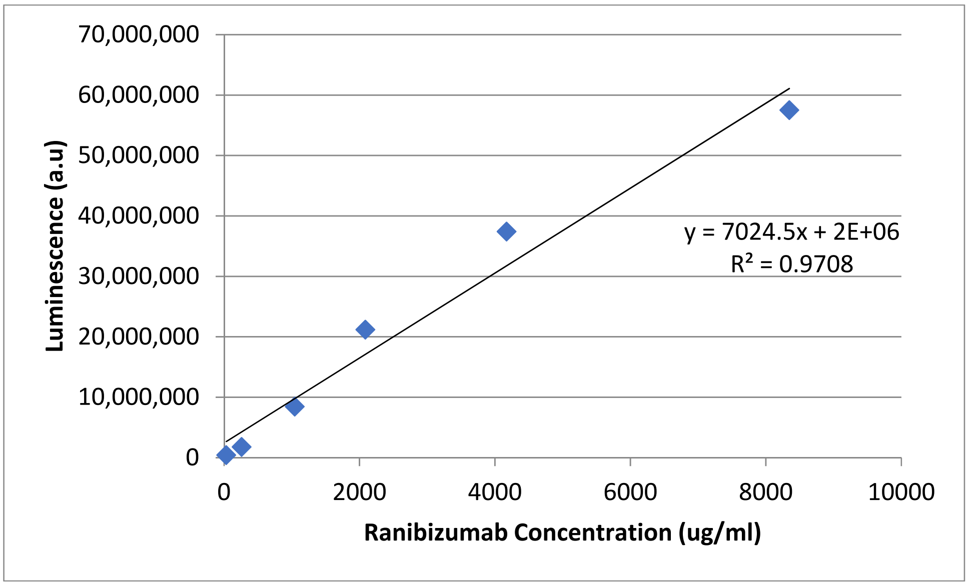

Figure S9. Standard curve of ranibizumab quantification through ELISA

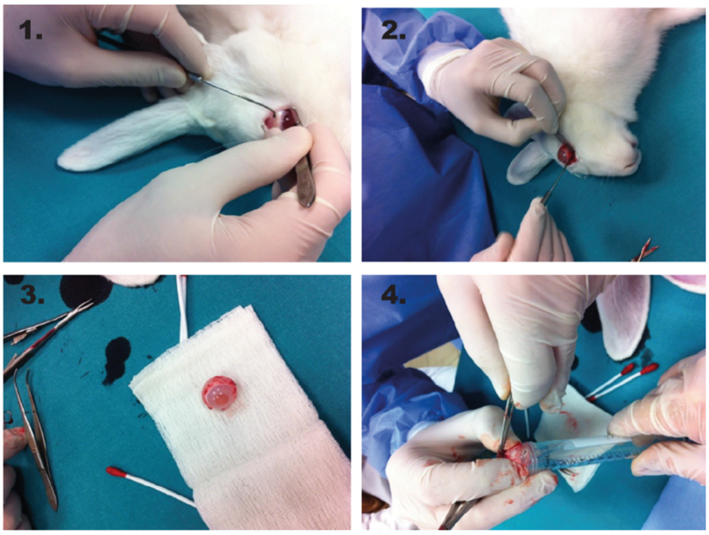

Figure S10. Phases of in vivo experiment.
